# The effects of season, geography, and urbanization on the diversity of edible insects at food markets in Laos

**DOI:** 10.1371/journal.pone.0267307

**Published:** 2022-04-18

**Authors:** Kazuki Tagawa, Tadatsugu Hosoya, Kimihiko Hyakumura, Dai Suzuki, Satoshi Yoshizawa, Bounthob Praxaysombath

**Affiliations:** 1 Institute of Decision Science for a Sustainable Society, Kyushu University, Fukuoka, Japan; 2 Graduate School of Systems Life Sciences, Kyushu University, Fukuoka, Japan; 3 Institute of Tropical Agriculture, Kyushu University, Fukuoka, Japan; 4 Graduate School of Social and Cultural Studies, Kyushu University, Fukuoka, Japan; 5 Faculty of Science, National University of Laos, Vientiane, Laos; Universidade Federal de Mato Grosso do Sul, BRAZIL

## Abstract

Laos, a mountainous and landlocked country located in Southeast Asia, has the highest percentage of people using insects as food in the world. Lao people obtain edible insects through harvesting in the natural environment and purchasing at food markets. There has been no comprehensive survey about sales of insects at food markets in the wider areas, and our understanding of sales of insects in Laos is limited. Our study aims to identify environmental factors affecting the sales and the diversity of edible insects sold at food markets in Laos. We visited 37 and 55 markets, during the dry and rainy seasons respectively, in northern Laos to record species of sold insects. We then analyzed the correlations between insect sales and three potential factors (seasons, provinces, and urbanization indices around the markets). There was no significant difference in the percentage of markets selling insects between in the dry and rainy seasons; 40–50% of the markets sold insects in both seasons. The composition of sold insects differed between in the dry and rainy seasons, which reflects the seasonality and life history of each insect species. There tended to be more groups of insects for sale in the Vientiane capital than in the other provinces in both seasons. This trend may reflect that it is more difficult to obtain edible insects through wild harvesting in highly urbanized Vientiane capital than in the other provinces, and the commercial demand for insects is increasing. This possibility is directly supported by the positive correlation between the urbanization index and the insect sales in the rainy season. Laos has recently undergone rapid urbanization, particularly in the Vientiane capital, and we predict that commercial demand for edible insects will be much higher in the Vientiane capital and the urbanized cities in the future.

## Introduction

By 2050, the world’s population is expected to reach 9.15 billion and the demand for meat is expected to increase by 76% from 2007 [1]. However, the expansion of cattle, pigs, and chickens production is not a sustainable option because it has negative impacts on the global environment: deforestation, soil erosion, desertification, loss of biodiversity, public health hazards, and water pollution [2]. In addition, given that livestock account for more than 14.5% of greenhouse gas emissions [3], the expansion of livestock production is likely to lead to increased global warming. Therefore, it is necessary to find alternative protein sources for cattle, pigs, and chickens. Insects are one of the candidates for alternative protein sources [2]. Some insects can be produced economically at a low-cost [4] and environmentally friendly [5] compared to livestock. Because of these features, the use of insects as foods (entomophagy) has attracted much attention as a new source of protein for a sustainable society.

Entomophagy is not new; its history dates back 7,000 years [6]. To date, more than 1,900 species of insects have been used as food, mainly in tropical and subtropical regions of Asia, Africa, and Oceania [2]. In tropical and subtropical regions, insects are relatively larger, have stable life histories, and are easy to collect, which have led to the development of insect-eating cultures [7].

In The Lao People’s Demographic Republic (Lao PDR, Laos), a mountainous and landlocked country located in Southeast Asia, a variety of wild animals have been collected and used as protein sources, including fish [8, 9], reptiles [10], birds [11], and mammals [12]. In addition, insects are one of the major protein sources; Laos has the highest percentage of people using insects as food in the world [13]. According to a national survey of 1,059 people living throughout the country, 98.8% had eaten insects in the past, 13.0% were daily or weekly consumers, and 31.1% were consumers a few times a month [14]. In Laos, each family has obtained edible insects mainly through wild harvesting [13, 15]. In addition, they also purchase edible insects at food markets; 34.9% of respondents in the national survey indicated that they were in the habit of purchasing edible insects [14]. Fragmentary studies of edible insects sold at food markets in Laos have been conducted in several regions (e.g., [15]). However, there has been no comprehensive survey at markets in wider areas, and our understanding of the sale of edible insects in Laos, an important aspect of entomophagy culture, is limited.

Given that many of the sold insects are collected in the wild [14, 16], the presence and diversity of edible insects sold at markets are expected to depend on the season and the surrounding natural environment. Under the influence of the monsoon, Laos has a rainy season from May to October, a cool dry season from November to February, and a hot dry season in March and April [17]. The life history of insects is seasonal even in tropical areas [18], and the species and life stages of insects available for collection vary with the season. Therefore, the diversity of insects sold at markets is expected to vary with the season. In addition, Laos has diverse wild habitats ranging from mountainous areas above 1,000 meters to plains along the Mekong River, and the insect species that can be collected differ depending on the habitats. The natural environmental conditions may also affect the diversity of edible insects sold at markets.

Insect abundance, biomass, and diversity are rapidly declining around the world [19, 20], which is no exception for edible insects (e.g., giant water bug in Thailand; [21]). One of the main drivers of the decline is habitat loss and degradation due to urbanization [22]. Urbanization removes plants from habitats to cause the decline of plant-associated insects (e.g., [23]), which can cascade the extinction of predators and parasitoids connected in the food web [22]. Urbanization also makes habitats of insects smaller, fragmented and isolated, making it difficult for insect populations to maintain over a long time [19]. In recent years, Laos has been undergoing rapid urbanization, especially in the Vientiane capital [24], which may lead to the loss of habitats suitable for edible insects, and make it difficult for collectors to harvest edible insects in the wild. Therefore, the level of urbanization around the market is likely to correlate negatively with the diversity of edible insects sold at markets.

The objective of this study is to investigate the diversity of edible insects sold at food markets in northern Laos to identify factors affecting the presence and the diversity of sold insects. Three hypotheses were formulated about potential factors. First, we expected that species compositions of sold insects would be different between in dry and rainy seasons. Second, we expected that species compositions of sold insects would vary depending on provinces differing in the condition of natural environments. Third, as the degree of urbanization increases in the vicinity of markets, the diversity of sold insects would decrease due to the difficulty in collecting insects.

## Materials and methods

### Study site

The Lao People’s Demographic Republic (Lao PDR, Laos) is a landlocked, mountainous country where ca. 58% of the country’s area is forest [25]. The northern part of the country is lined with mountains over 1,000 meters high, while the elevation around the Vientiane capital is only about 100 meters. The country is surrounded by Cambodia, China, Myanmar, Thailand, and Vietnam. More than two-thirds of the country’s 7 million people live in rural areas and earn their living mainly through agriculture. The development of the road network and the increase in the number of people owning cars has led to an increase in automobile-mediated exchanges between regions.

**Fig 1:**
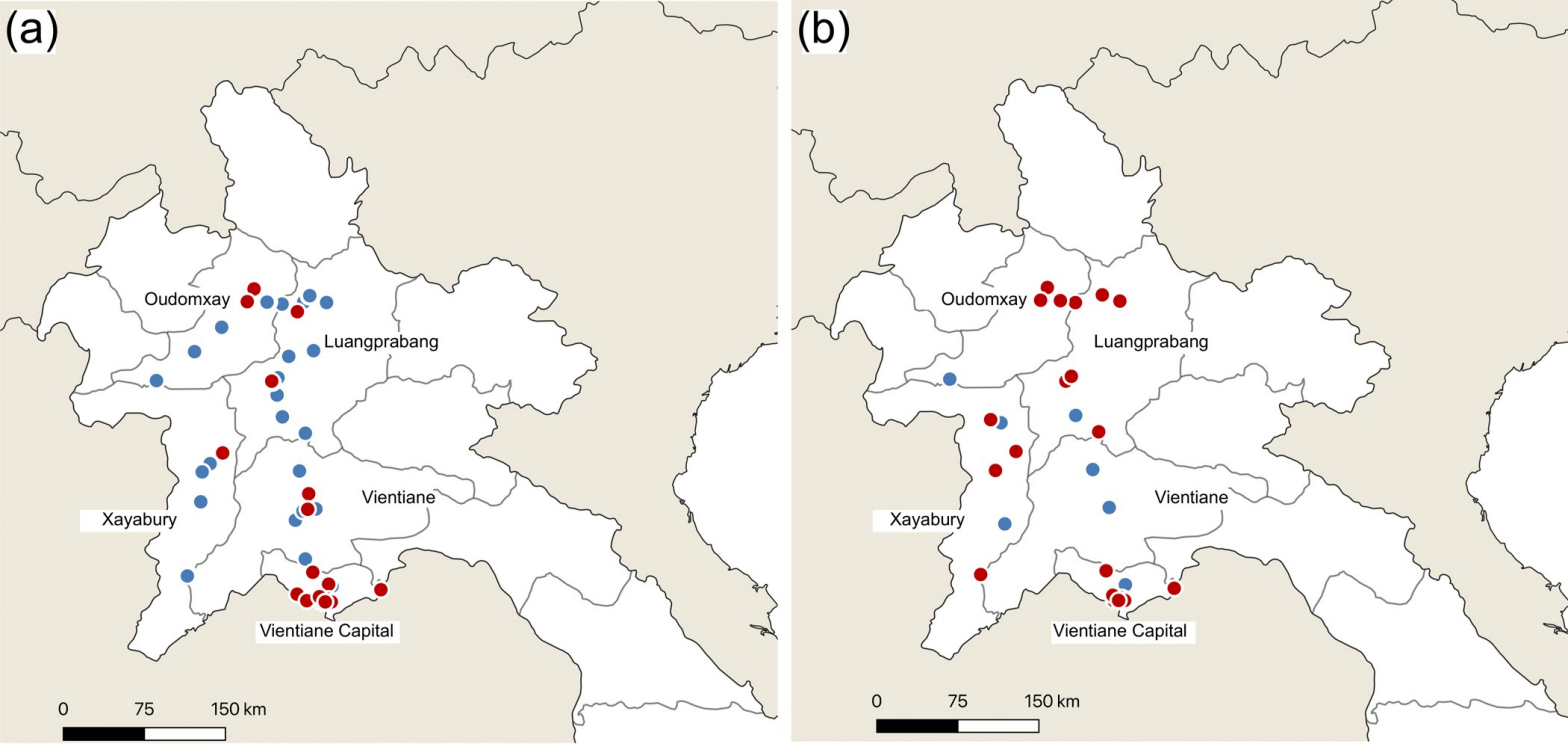
Geographical locations of food markets under observations in Laos. (a) dry season, (b) rainy season. Red circles indicate markets where at least one species of edible insects was observed. Blue circles indicate markets where no edible insect was observed.

We conducted the surveys at food markets located in the Vientiane capital, Vientiane Province, Luang Prabang Province, Oudomxai Province, and Xaignabouri Province in northern Laos (Fig 1). In recent years, foreign investment in infrastructure has increased, and the Vientiane capital is undergoing rapid urbanization [24, 26]. From 1995 to 2015, the built-up area in the Vientiane capital has increased from 3.4% to 12.81% [27]. The plains around the city are covered with rice paddies and marshlands [28]. The southern part of Vientiane Province is dominated by farmlands. In Luang Prabang Province, many people have migrated from rural areas to cities, and urbanization is progress in central cities [29]. The northern part of Luang Prabang Province, Oudomxai Province, and Xaignabouri Province, are mostly mountainous.

There is at least one food market in all the towns in Laos [30]. In the food markets, in addition to edible insects, plants, mushrooms, seafood, and meats are also sold [15, 30].

### Investigation methods

**Fig 2:**
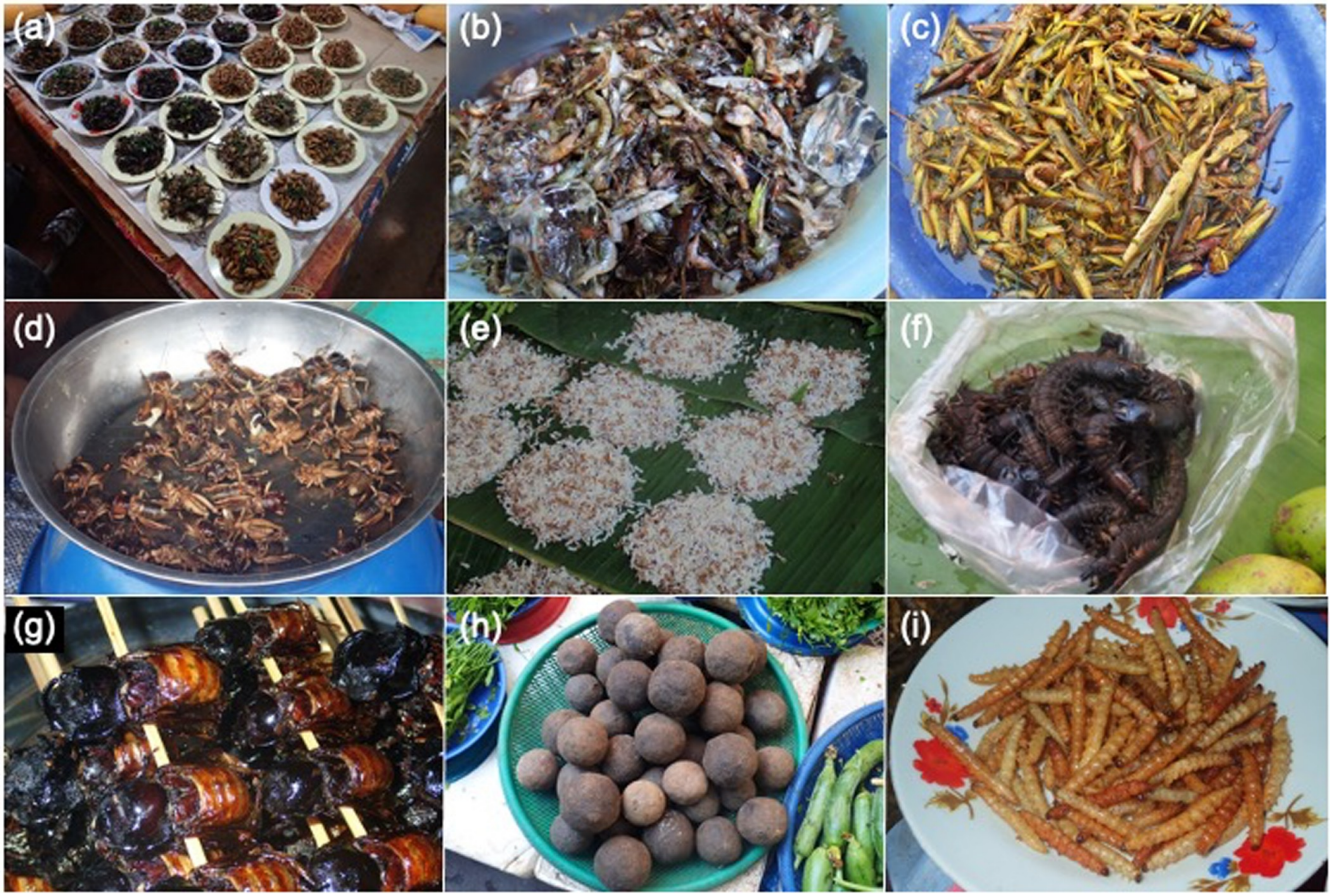
Edible insects observed at food markets in Laos. (a) Insects are usually classified roughly and sold on one dish. (b) Dragonfly nymphs are sold together with freshwater fish and shrimps. (c) Adults and nymphs of grasshoppers (*Atractomorpha* sp., *Acrida* spp., *Euconocephalus* sp., etc.). (d) Adults of short-tailed crickets (*Brachytrupes portentosus*). (e) Larvae, pupae, and adults of weaver ants (*Oecophylla smaragdina*). (f) Larvae of dobsonflies (Family Corydalidae). (g) Fried rhinoceros beetles (*Trichogomphus martabani*), (h) Dung balls of elephant dung beetle (*Heliocopris bucephalus*). (i) Fried bamboo caterpillars (*Omphisa fuscidentalis*).

From September 3^rd^ to 13^th^ 2015 (rainy season), we visited 37 food markets. From February 4^th^ to 13^th^ 2017 (dry season), we visited 55 food markets. We walked through each market and recorded whether edible insects were sold or not. Sold insects were photographed with a digital camera. The status of the insects at the time of sale (i.e., alive, dead and uncooked, or cooked) was also recorded. Insect species observed for the first time were purchased and specimens were preserved in 95% ethanol. Multiple species of insects were often sold together without sorting on a single plate (Fig 2A); for example, *Atractomorpha* spp., *Acrida* sp., *Euconocephalus* spp., and *Locusta migratoria* were sold together as "grasshoppers" (Fig 2C). Since the purpose of this study was not to make a detailed list of sold insects, but rather to identify the potential factors that influence the sales of edible insects, we tabulated the data for 23 functional groups, which were often sold together without sorting on a single plate, in reference to previous studies about edible insects in Laos [2, 13]. Latitude, longitude, and elevation of the markets were recorded using GPS. The area of the market was also measured based on aerial photographs from the Laos national forest monitoring system [31]. The area was measured as the entire building area, excluding the parking lot. In addition, as an indicator of urbanization around the market (urbanization index), we drew a circle with a radius of 1 km around the market and visually judged the percentage of buildings occupying the circle, and recorded the results in five levels (0: <10%, 1: 10–30%, 2: 30–50%, 3: 50–70%, 4: 70–100%).

### Statistical analyses

To determine whether the percentage of markets where edible insects were sold differed between in the dry and rainy seasons, the percentage of markets where one or more insects were sold and the percentage of markets where sales were confirmed for each group of insects were compared using Fisher’s exact probability test. A generalized linear model (GLM) analysis was conducted to determine the potential factors affecting insect sales at food markets. The response variables were whether insects were sold (1/0; binary) or the number of groups of edible insects, and the explanatory variables were the province/capital of the market, urbanization index, elevation, and market area. When the response variable was whether the insects were sold, the error structure was a binomial distribution and the link function was logit. When the response variable was the number of groups of edible insects, the error structure was a Poisson distribution and the link function was log. For each of the dry and rainy season, a total of 24 models were computed and examined for the overall and each insect group’s respective sales. Multicollinearity among explanatory variables was checked based on the VIF statistic; VIF < 10 for each variable in all models, indicating that the effect of multicollinearity was small. Significance was confirmed using the likelihood ratio test. All statistical analyses were performed using R [32] and the package "car" [33].

## Results

We identified sales of Odonata, Orthoptera, Coleoptera, Hemiptera, Hymenoptera, Lepidoptera, and Megaloptera (Table 1). The percentages of markets selling at least one species of insects were 41.8% (23/55) in the dry season and 46.0% (17/37) in the rainy season respectively, with no statistically significant difference between the seasons (Table 1, Fisher’s exact probability test, *P* = 0.42).

**Table 1:** The percentages of food markets selling each group of insects in dry and rainy seasons respectively.

Popular name	Scientific name	Dry season (%)	Rainy season (%)	*P* value
Dragonfly nymphs (Fig 2B)	Order Odonata	25.5	8.6	*
Grasshoppers (Fig 2C)	*Atractomorpha* sp., *Acrida* spp., *Euconocephalus* sp., *Locusta migratoria*, etc.	9.1	8.6	NS
Short-tailed crickets (Fig 2D)	*Brachytrupes portentosus*	7.3	28.6	**
Domestic house crickets	*Acheta domesticus* ^#^	9.1	22.9	NS
Mole crickets	*Gryllotalpa africana*	1.8	0	NS
Other crickets	Family Gryllidae	12.7	0	*
Stink bugs	*Tessaratoma* sp.	3.6	2.9	NS
Giant water bugs	*Lethocerus indicus*	9.1	2.9	NS
Honey bees	*Apis* spp.	10.9	2.9	NS
Wasps	*Vespa affinis*	0	17.1	**
Weaver ants (Fig 2E)	*Oecophylla smaragdina*	21.8	0	**
Subterranean ants	*Carebara castanea*	16.4	0	*
Dobsonflies (Fig 2F)	Family Corydalidae	1.8	0	NS
Rhinoceros beetles (Fig 2G)	*Trichogomphus martabani*	0	11.4	*
Five-horned rhinoceros beetles	*Eupatorus gracilicornis*	0	5.7	NS
Brown rhinoceros beetles	*Xylotrupes gideon*	1.8	17.1	**
Elephant dung beetles (Fig 2H)	*Heliocopris bucephalus*	1.8	0	NS
Palm weevils	*Rhynchophorus ferrugineus*	1.8	0	NS
Diving beetles and water scavenger beetles	Family Dytiscidae Family Hydrophilidae	5.5	0	NS
Bamboo caterpillars (Fig 2I)	*Omphisa fuscidentalis*	5.5	31.4	***
Silk worms	*Bombyx mori* ^#^	10.9	17.1	NS
Other moths	Order Lepidoptera	3.6	5.7	NS
	Percentage of markets that at least one species of insects was sold	41.8	46.0	NS

Fisher’s exact probability test, NS: Not significant

* *P* < 0.05

** *P* < 0.01

*** *P* < 0.001.

^#^ Mainly acquired by farming.

There was a difference in the compositions of insect groups identified between the dry and rainy seasons (Table 1). Crickets (Family Gryllidae), mole crickets (*Gryllotalpa africana*), weaver ants (Fig 2E, *Oecophylla smaragdina*), ants (*Carebara castanea*), dobsonflies (Fig 2F, Family Corydalidae), and palm weevils (*Rhynchophorus ferrugineus*) were found for sale only in the dry season. For dragonfly nymphs (Fig 2B, Family Odonata), the percentage of markets with sales was significantly higher in the dry season than in the rainy season (Fisher’s exact probability test, *P* < 0.05). Sales of rhinoceros beetles (Fig 2G, *Trichogomphus martabani*), five-horned rhinoceros beetles (*Eupatorus gracilicornis*), and wasps (*Vespa affinis*) were observed only in the rainy season. For bamboo caterpillars (Fig 2I, *Omphisa fuscidentalis*), short-tailed crickets (Fig 2D, *Brachytrupes portentosus*), and brown rhinoceros beetles (*Xylotrupes gideon*), the percentages of markets with sales were significantly higher in the rainy season than in the dry season (Fisher’s exact probability test, *P* < 0.05). The percentage of markets where domestic house crickets (*Acheta domesticus*) were sold tended to be higher in the rainy season compared to the dry season (Fisher’s exact probability test, *P* = 0.07). There were no significant differences in the percentages of markets selling giant water bugs (*Lethocerus indicus*), stink bugs (*Tessaratoma* sp.), honey bees (*Apis* spp.), silk moths (*Bombyx mori*), grasshoppers (Fig 2C) between the seasons.

**Fig 3:**
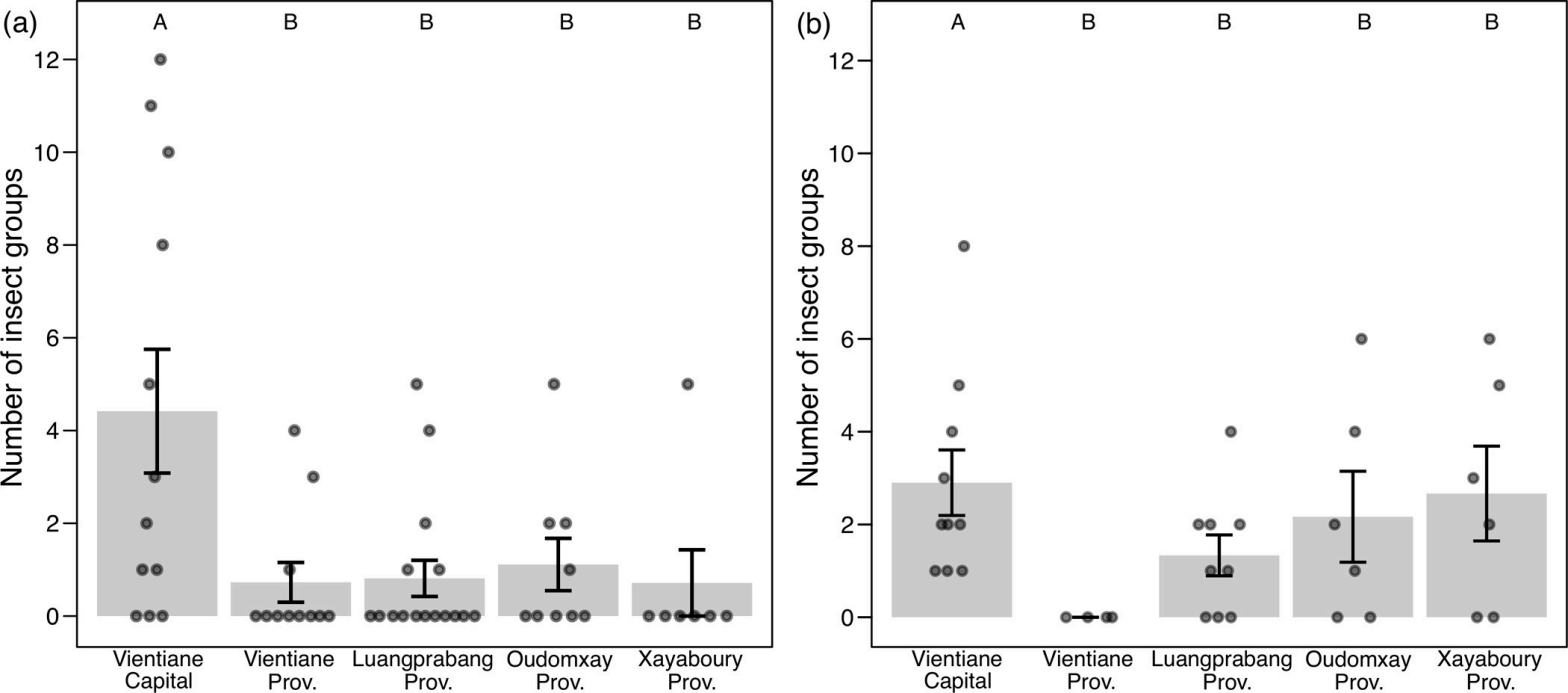
The number of groups of insects sold at food markets in each province and capital. (a) Dry season, (b) Rainy season. Circles indicate the number of insect groups sold at each market. Bars and whiskers indicate the mean and SD for each province and capital respectively. Letters above the figure indicate significant differences at 5% level (generalized linear models with Holm adjustments).

**Fig 4:**
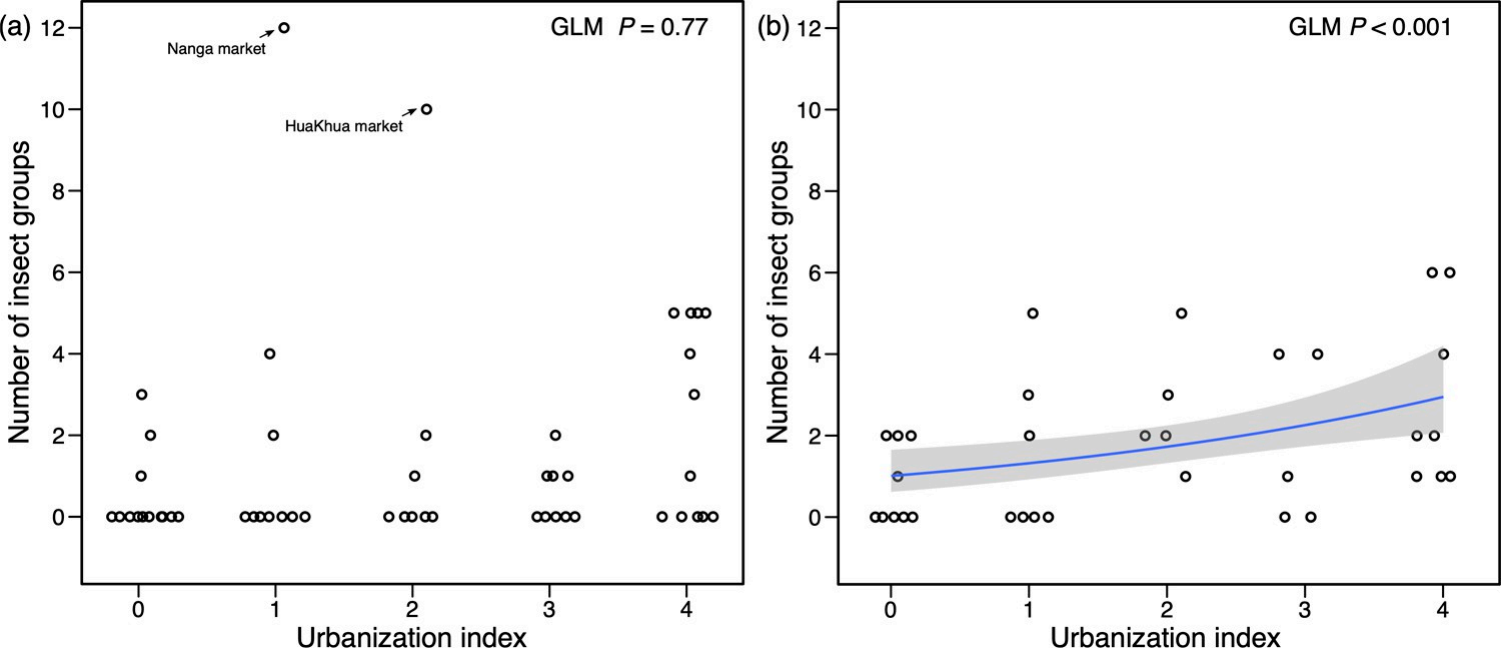
Relationship between the number of groups of insects sold at food markets and urbanization index. (a) Dry season, (b) Rainy season. Circles indicate the number of insect groups sold at each market. Blue line represents the fitted curve by a generalized linear model with Poisson distribution (log link function).

In the dry season, there was no statistically significant effect of province/capital, urbanization index, elevation, or market area on the percentage of markets with at least one insect group for sale. The number of groups of insects identified in markets differed significantly by province/capital (GLM, χ^2^ = 28.4, *P* < 0.001), but the effects of urbanization index, elevation, and market area were not significant. We made a model that the data of silk moths (*B*. *mori*) and domestic house crickets (*A*. *domesticus*), which are mainly acquired through farming, were excluded, and confirmed that these explanatory variables were not significant. The number of insect groups was significantly higher in the Vientiane capital than in the other provinces (Fig 3A). The percentage of markets selling silk moths (*B*. *mori*) (GLM, χ^2^ = 28.4, *P* < 0.01), weaver ants (*Oecophylla smaragdina*) (GLM, χ^2^ = 12.7, *P* < 0.05), and dragonfly nymphs (GLM, χ^2^ = 14.9, *P* < 0.01) also differed significantly across province/capital, with a common higher percentage in the Vientiane capital compared to the other provinces. There was no significant effect of the urbanization index on the number of insect groups. However, excluding the data from two markets (i.e., Hua Khua market [number of groups: 10, urbanization index: 2]; Nanga market [number of groups: 12, urbanization index: 1]), the number of insect groups and urbanization index showed a significant positive correlation (GLM, χ^2^ = 14.7, *P* < 0.001, Fig 4A).

During the rainy season, the percentage of markets with at least one group of insects on sale differed significantly across province/capital (GLM, χ^2^ = 10.7, *P* < 0.05), and the higher the urbanization index, the significantly higher the percentage of markets with insects on sale (GLM, χ^2^ = 4.29, *P* < 0.05). The number of groups of insects sold differed significantly among province/capital (GLM, χ^2^ = 22.1, *P* < 0.001). The higher the urbanization index, more groups of insects were sold (GLM, χ^2^ = 4.14, *P* < 0.05; Fig 4B). Elevation and market area had no significant effect on the percentage of markets where insects were sold and on the number of insect groups. In the Vientiane capital, the percentage of markets where insects were sold was 100%, while in Vientiane province it was 0%. In other provinces, there was no significant difference at around 60%. The number of groups of insects sold was significantly higher in the Vientiane capital than in the other provinces (Fig 3B). The percentage of markets selling domestic house crickets (*A*. *domesticus*) (GLM, χ^2^ = 11.4, *P* < 0.05) and rhinoceros beetles (*T*. *martabani*) (GLM, χ^2^ = 9.46, *P* = 0.05) were also significantly different among province/capital. The percentage of bamboo caterpillars (*O*. *fuscidentalis*) (GLM, χ^2^ = 5.73, *P* < 0.05) were sold increased with higher urbanization indices. Rhinoceros beetles (*T*. *martabani*) tended to be sold in markets with smaller areas (GLM, χ^2^ = 8.90, *P* < 0.01).

## Discussion

### Seasonality in groups of insects sold at food markets

There was no significant difference in the percentage of markets selling insects between in the dry and rainy seasons; insects were sold in 40–50% of the markets in both seasons. The composition of sold insects differed between in the dry and rainy seasons, which reflects the seasonality and life history of each insect group. In particular, for insect groups that are used as foods on specific life-history stages, such as eggs, larvae, nymphs, pupae, and adults, as food, sales were confirmed only during the seasons when those stages were observed. For example, bamboo caterpillars (*O*. *fuscidentalis*) exhibit annual life cycles, with adults emerging in July and August, laying eggs on bamboo shoots in early August, followed by the emergence of larvae [13]. Since only the larvae are edible, their sales were observed only during the rainy season (September 2015) when the larvae can be caught. Similarly, for short-tailed crickets (*B*. *portentosus*), rhinoceros beetles (*T*. *martabani*), five-horned rhinoceros beetles (*E*. *gracilicornis*), and wasps (*V*. *affinis*), which exhibit an annual life cycle and have specific developmental stages for food use [13], sales were confirmed only during the rainy season, and sales of weaver ants (*O*. *smaragdina*) were confirmed only during the dry season. The seasonality of these sales was consistent with previous studies that examined insect use as food in Laos [13]. In contrast, for aquatic dragonfly nymphs, seasonality of sales was not consistent with the seasonality of insect use as food in Laos described in previous studies; their sales were more often observed during the dry season compared to the rainy season, while previous studies showed that harvesting of dragonfly nymphs occurs throughout the year and the peak of harvesting is during the rainy season [13]. One possible reason for the difference is that during the rainy season, many temporary water places suitable for dragonfly nymphs to habit are formed, and as a result of the ease with which local people can capture them in their immediate surroundings, the demand for them in markets may become lower. This possibility will be tested through interviews with buyers and sellers.

### The effects of geographical factors and the urbanization on groups of edible insects sold at food markets

We expected a negative correlation between the urbanization index and the diversity of edible insects sold at markets. However, the findings were the opposite; in the rainy season, there was a positive correlation between the urbanization index and the diversity of edible insects. In addition, in both dry and rainy seasons, there tended to be more groups of insects for sale in the urbanized Vientiane capital than in the other provinces. From these findings, we can point out two suggestions about the effects of urbanization on the sales of edible insects in Laos. First, even in urbanized cities, collectors have access to edible insects. However, it may be becoming more difficult to collect insects than before. Boulidam (2010), who studied the availability of edible insects at markets in the Vientiane capital, reported that the number of collectors was increasing and collectors needed more time to harvest enough amounts of edible insects nowadays [15]. Second, in urban areas, the commercial demand for edible insects of people is high. This may reflect the situation that it is difficult for local people to capture insects by themselves in urban areas. Even in the urbanized Vientiane capital, the percentage of people using insects as food frequently was over 80% [14]. On the other hand, farmlands and forests, which are the main fields for harvesting edible insects [16], have been reclaimed, and the proportion of houses, stores, and factories has been increasing [24], making it difficult to get edible insects in the wild. Furthermore, with the modernization of life, people do not have enough time for wild harvesting [24]. Therefore, the commercial demand for edible insects is likely to be higher in urban areas. In our study, the positive correlation between the urbanization index and insect diversity was found only in the rainy season and not in the dry season. According to an interview survey to sellers [14], 88% of sellers felt that it was more difficult to sell insects during the rainy season than the dry season because it was easier for people to harvest insects by themselves in the rainy season, and they tended to get insects not by purchasing but by wild harvesting. Therefore, the commercial demand of people in urbanized areas may be more evident in the rainy season than in the dry season.

Given that urbanization is expected to continue, the commercial demand for edible insects in the Vientiane capital and other major cities may be even higher in the future. In addition, the FAO’s promotion of edible insects as a source of protein in Laos [13], may also facilitate the demand for insects as foods. The over-harvesting of insects by collectors due to increasing commercial demand in urban areas may reduce the population sizes of edible insects in suburban areas, resulting in either local or total extinction of insects [21]. It will also lead to difficulties in securing food for local people who harvest insects as a subsistence diet. In fact, due to the increase in the number of collectors and the decrease in the forest area, the number of a popular edible insect species, weaver ant (*O*. *smaragdina*) has been decreasing, making it difficult for local people to collect them [34]. Considering that insects are a valuable source of protein, vitamins, and minerals for local people, the negative impact of difficulty in collecting edible insects for subsistence use on food security is significant. There are three ways to reduce the chance of decrease and local extinction of edible insects in the wild: 1: managing wild harvesting, 2: farming, and 3: managing consumer experience [21]. In order to effectively manage wild harvesting, it is important to collect scientific knowledge on the ecology of edible insects and traditional ecological knowledge about harvesting edible insects from local people. Local people monitor the development of edible insects to determine the appropriate time and method of harvesting for sustainable use [35, 36]. In Laos, observations on collection practices and semi-structured interviews of local people harvesting weaver ants suggested that they intentionally refrained from collecting the majority of their nests, which led to the maintenance of ant populations [34]. Research on sustainable insect use by local people in Laos is scarce compared to other regions, and further studies are needed. The cultivation of domestic house crickets has recently started in Laos through technology transfer from Thailand [13]. FAO has conducted training courses to teach farmers breeding techniques, insect marketing, and the nutritional values of the major four edible insects (i.e., domestic house crickets, mealworms, palm weevils, and weaver ants) and some farmers are now cultivating these insects [13]. There is a possibility that local insect species such as grasshoppers and other crickets can be cultivated in a similar way to domestic house crickets, and further research is desirable.

### Novel use of edible insects in Laos

In this study, to the best of our knowledge, the food use of dobsonflies (Corydalidae) was confirmed for the first time in Laos. In the dry season, larvae of dobsonflies were sold at a market in one mountainous area in Oudomxay province. The larvae of dobsonflies inhabit running water environments and are used as food in mountainous areas in China, Japan, Peru, and Mexico [37]. The use of natural resources for food in mountainous areas has not been fully elucidated, and insects inhabiting unique habitats are likely being used as unknown food. It is desirable to continue field research from a cultural anthropological perspective.

### Limitations and future research directions

This study revealed the relationship between seasonal/geographical factors and sales of edible insects at food markets in northern Laos. Some insect species were not found for sale even though they were reported as major edible insect species in Laos (e.g., cicada nymphs; [13]). This could reflect the difference between the insect species that are purchased for sale and those that people harvest by themselves around their homes. However, we should be cautious in interpreting this. Our survey was conducted only once for each market in each season, so we may not have a comprehensive picture of the diversity of insects that are collected and brought in by accident. To understand the relationships between seasonal/geographical factors and sales of edible insects more accurately, it is necessary to conduct surveys over multiple days and hours at a single market.

## Supporting information

S1 Data (XLSX)
